# Supplementary material for: Evolution of immune responses to SARS-CoV-2 in mild-moderate COVID-19
Source: Nat Commun. 2021 Feb 19;12:1162. doi: 10.1038/s41467-021-21444-5 (PMC7896046; doi:10.1038/s41467-021-21444-5)
Supplement: Supplementary file 1 — Supplementary informations [file 41467_2021_21444_MOESM1_ESM.pdf]

Supplementary Information

Evolution of immune responses to SARS-CoV-2 in mild-moderate COVID-19

Wheatley *et al*

Supplementary Table 1. Demographic and clinical characteristics of the uninfected and convalescent COVID-19 cohorts.

	Full Cohort (n=64)	Uninfected Serological Cohort (n=32)	Cellular Analysis Cohort (n=31)	Uninfected Cellular Analysis Cohort (n=20)
Age, median (IQR)	55 (49, 62)	53 (28, 60)	52 (31, 56)	51.5 (26, 58)
Gender, % female (n)	43.8% (28)	53.1% (17)	45.2% (14)	45% (9)
Disease severity, % (n)<br>- mild	68.8% (44)	-	74.2% (23)	-
- moderate	23.4% (15)	-	16.1% (5)	-
- severe	7.8% (5)	-	9.7% (3)	-
Positive PCR test, % (n)	84.4% (54)	-	83.9% (26)	-

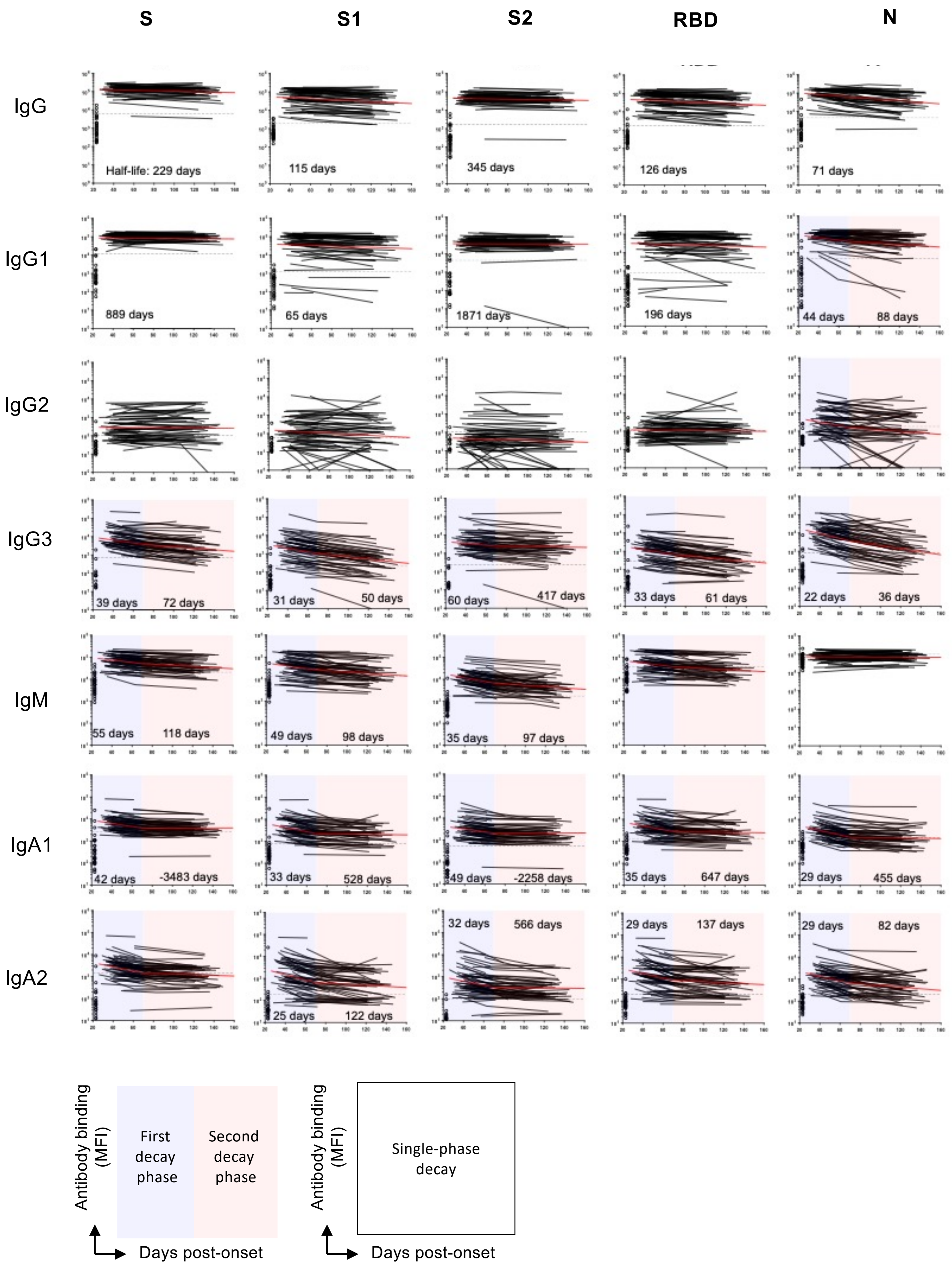

Supplementary figure 1: Fitting of the decline in antibody binding across different immunoglobulin isotypes.

Supplementary figure 1: Fitting of the decline in antibody binding across different immunoglobulin isotypes.

The best-fit model and half-lives are shown for the fitting of the decay of antibody binding to different SARS-CoV-2 antigens (n=64 participants). Two-phase decay is indicated by red (before day 70) and blue (after day 70) shaded areas. No shading indicates where single-phase decay provided the best fit. Uninfected control participants (n=32) are shown on the left side of each graph and horizontal dashed lines indicate the 90th percentile value of the uninfected control cohort. Note that for IgG2 to all antigens, IgM, IgA1 and IgA2 to N, IgM to RBD, and IgA2 to S, the SARS-CoV-2 infected cohort has a sizeable proportion (>25%) of responses at the first time point that are below the 90th percentile of the 32 uninfected controls and we have not calculated decay half-lives for these responses. Source data are provided as a Source Data file.

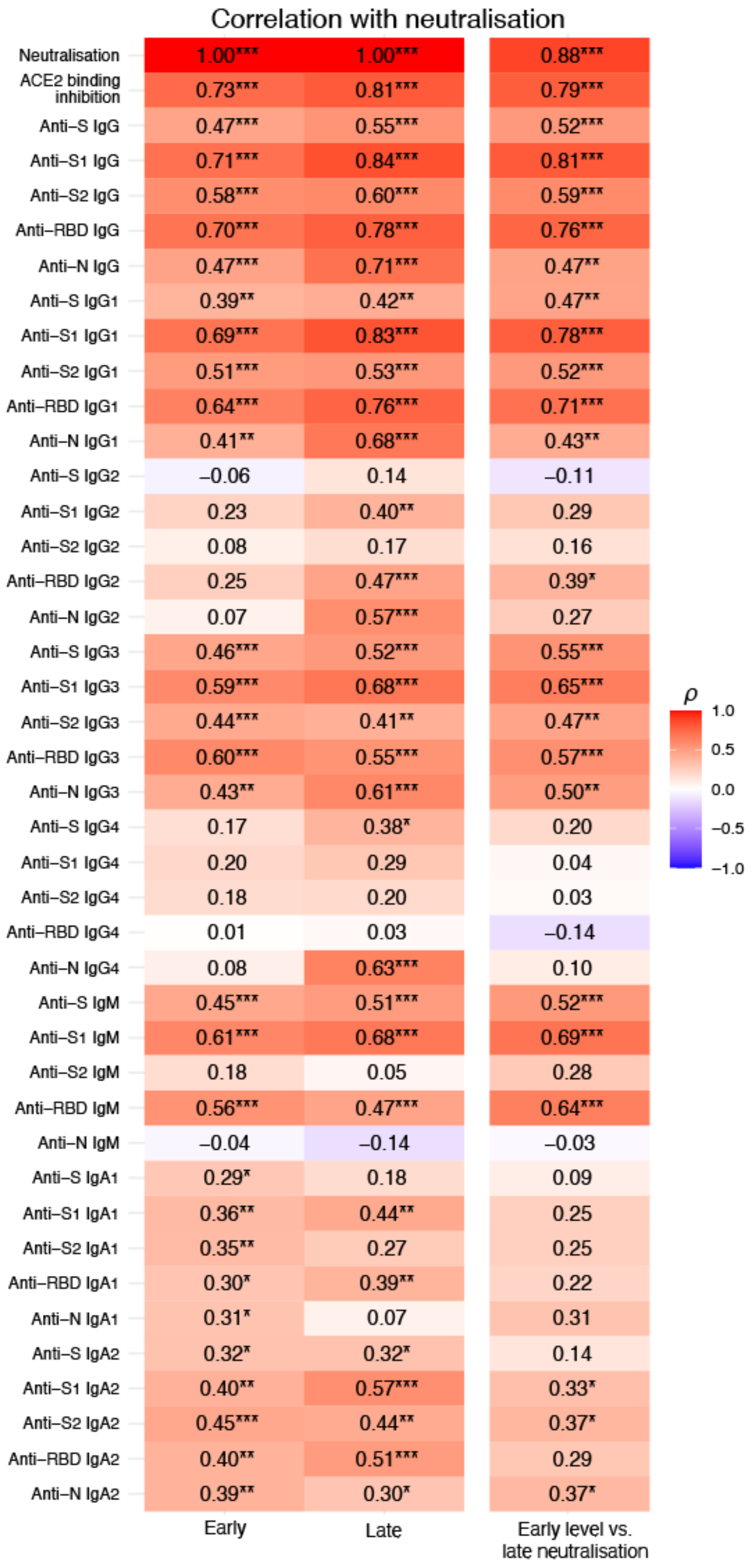

Supplementary figure 2: Correlation of antibody binding and ACE2 inhibition with neutralisation.

A heat-map of Spearman correlations between neutralisation titre and the serological measurements of antibody binding (by isotype and antigen). Correlations were assessed in early (≤50 days, left column n=54 participants) and late (≥100 days, right middle column, n=47 participants) convalescence in all participants were data was available. The association between early antibody binding and late neutralisation is also shown (right column, n=47 participants). All correlations are Spearman correlations. *P = 0.01-0.05, **P = 0.001-0.01, ***P≤0.001.

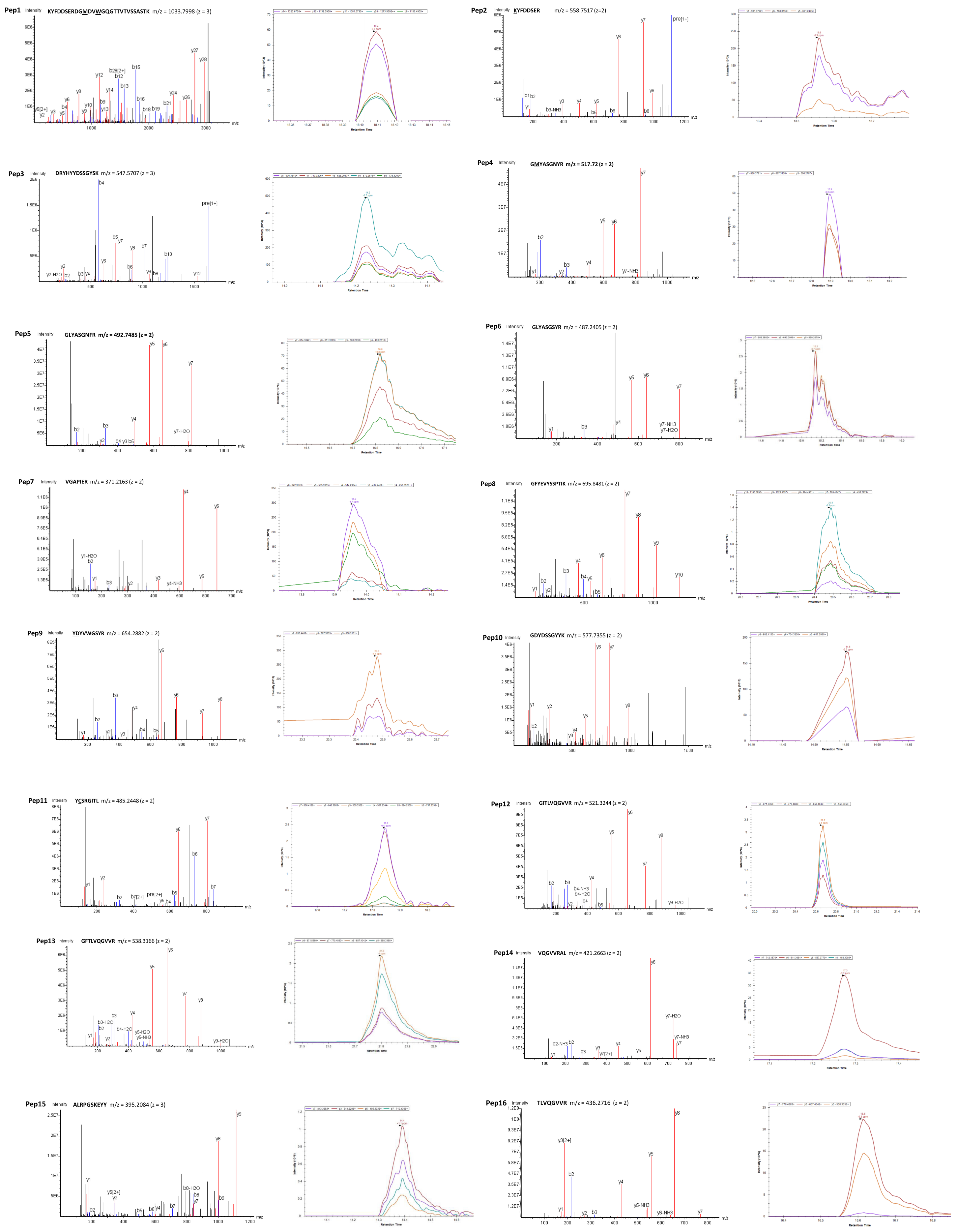

Supplementary Figure 3: Representative annotated MS/MS spectra (left panel) and their corresponding extracted ion chromatograms (XICs; right panel).

Supplementary Figure 3: Representative annotated MS/MS spectra (left panel) and their corresponding extracted ion chromatograms (XICs; right panel). The peptides used in PRM analyses are the matched clonotypic CDR-H3 peptides (pep1-16). Underlined amino acid indicates a post translational modification: M (oxidised methionine), W (oxidised tryptophan), K (carbamidomethylated lysine), D (carbamidomethylated aspartate), C (carbamidomethylated cysteine), and Y (acetylated tyrosine). The sequences, m/z and z of each individual peptides are shown on the top of their annotated MS/MS spectra. Matched b ions are indicated in blue and y ions are in red. m=mass, z=charge. MS raw data are available upon request.

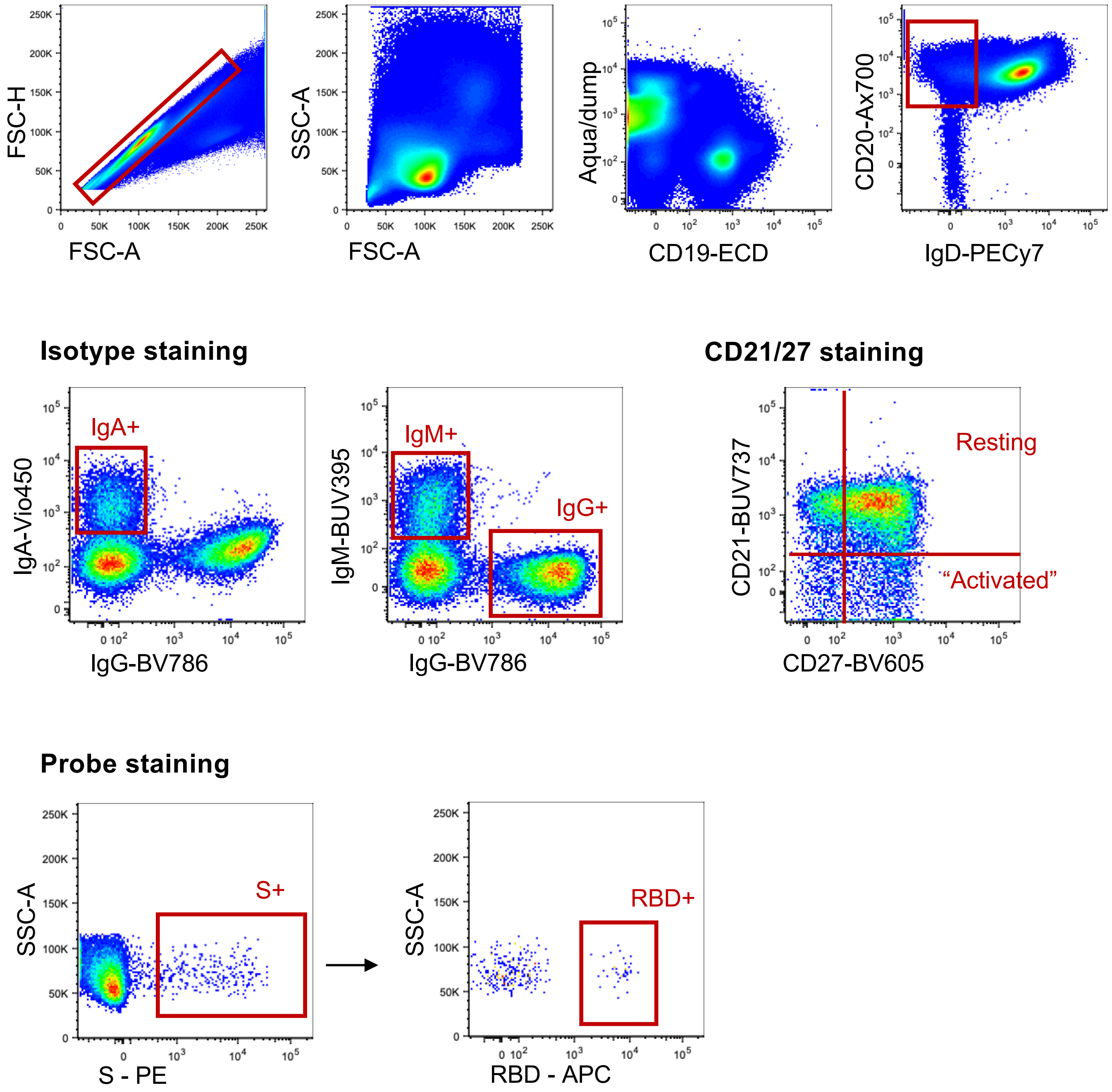

Supplementary Figure 4: Gating strategy for resolving antigen-specific B cells and surface isotypes.
After doublet exclusion (FSC-A vs FSC-H) and lymphocyte gating (FSC-A vs SSC-A), live CD19+IgD-CD20+ B cells were gated based on surface immunoglobulin expression (IgM, IgG, IgA). Binding to SARS-CoV-2 spike (S) and/or SARS-CoV-2 RBD probes was assessed for each population. Memory B cell phenotypes were identified by CD21 and CD27 co-staining.

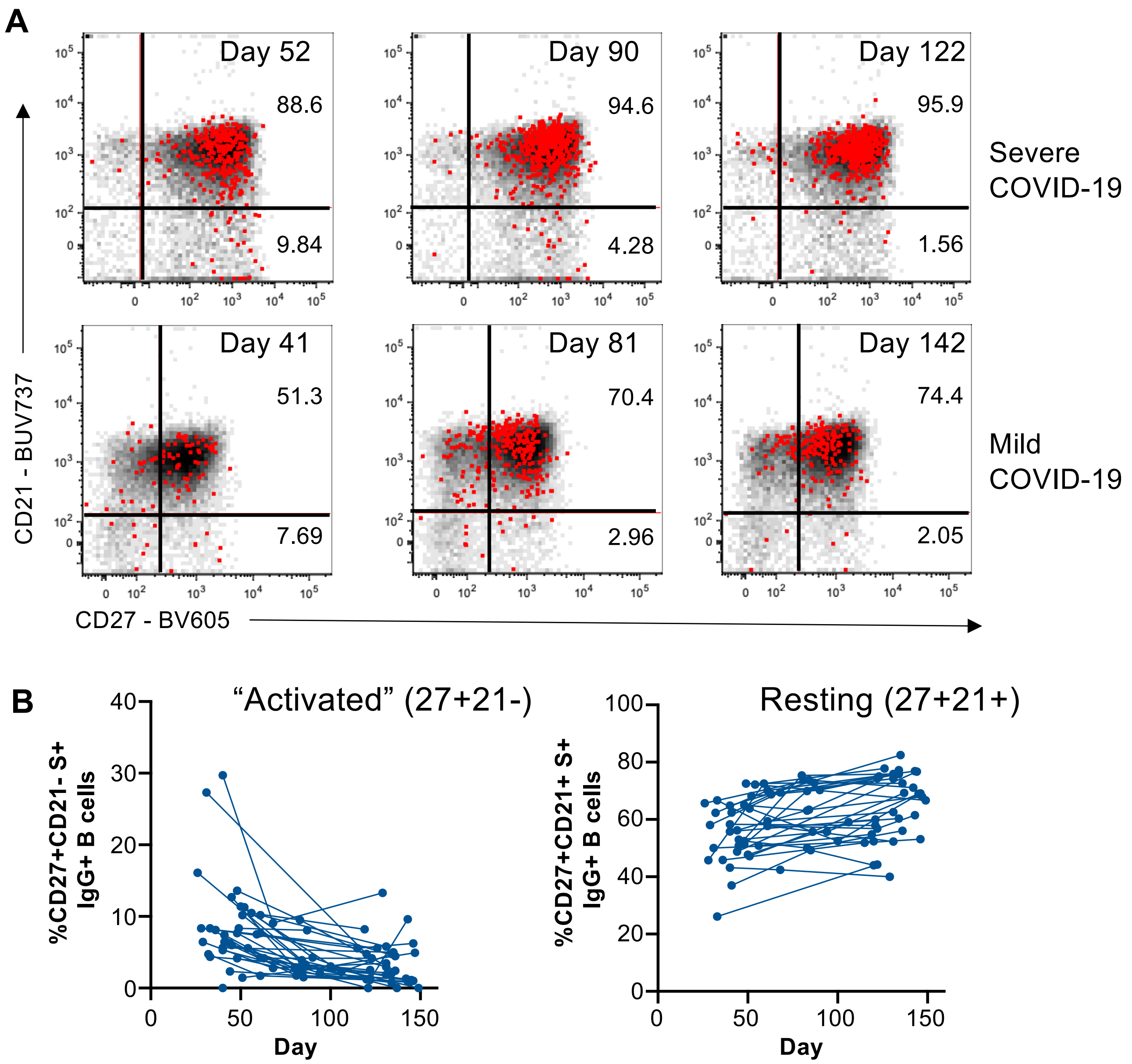

Supplementary Figure 5: Activation status of S-specific IgG+ memory B cells.

(**A**) Memory B cell phenotypes identified by CD21 and CD27 co-staining of S+CD19+CD20+IgD-IgG+ B cells (red) overlaid onto parental CD19+CD20+IgD-IgG+ B cells (black) and (**B**) the corresponding frequencies of "activated" (CD27+CD21-) or resting (CD27+CD21+) in in PBMC samples were assessed longitudinally (n=31 participants). Source data are provided as a Source Data file.

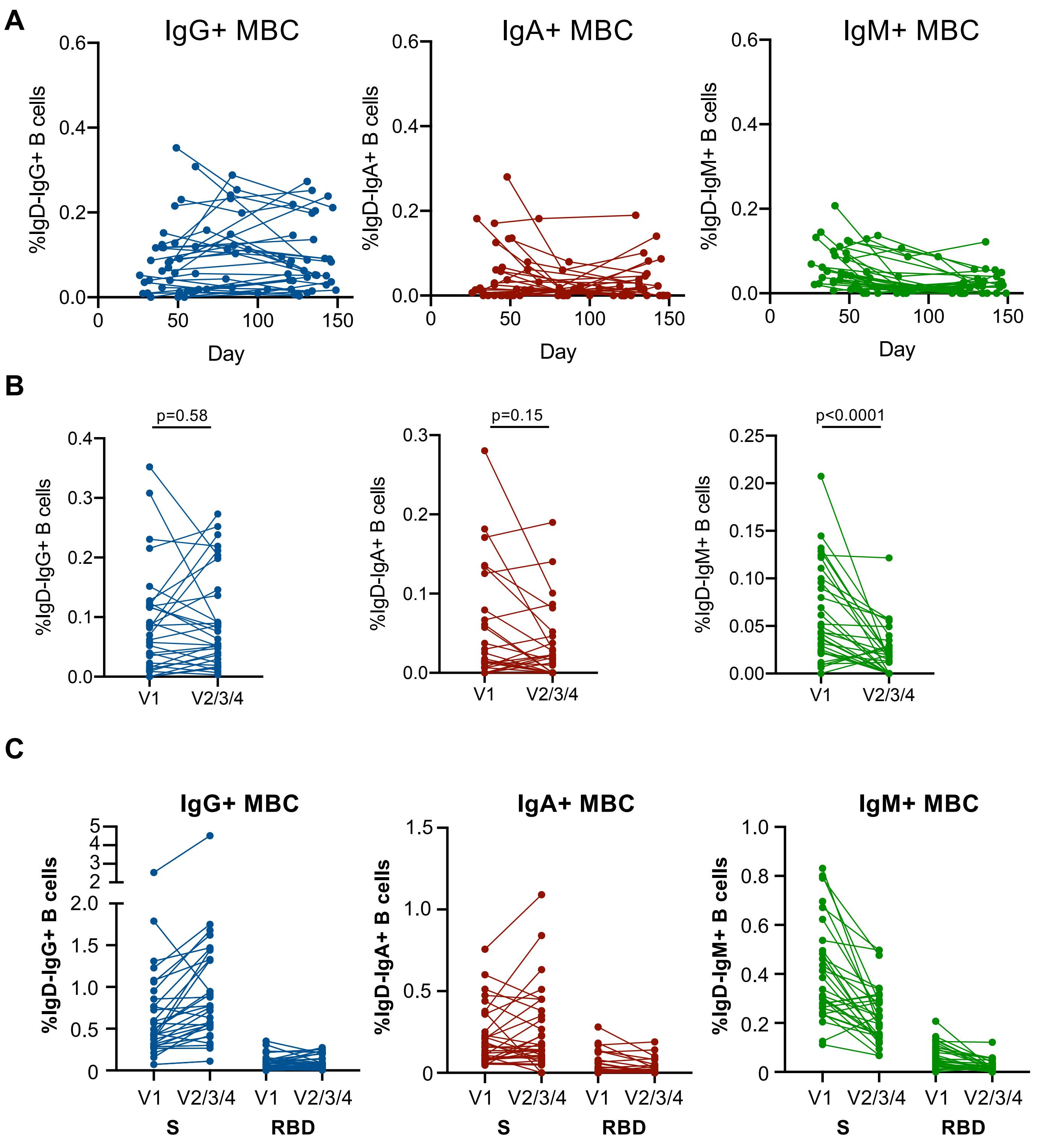

Supplementary Figure 6: RBD-specific memory B cell dynamics.

(**A**) Frequencies of RBD-specific IgG+, IgA+ or IgM+ memory B cells as a proportion of CD19+CD20+IgD- B cells in PBMC samples were assessed longitudinally. (**B**) Comparison of RBD-specific IgG+, IgA+ or IgM+ memory B cell frequencies at the earliest and latest timepoint available for each individual (n=31). Statistics assessed by two-tailed Wilcoxon test. (**C**) Direct comparison of S- and RBD-specific memory B cell frequencies at the earliest and latest timepoint available for each individual (n=31). Source data are provided as a Source Data file.

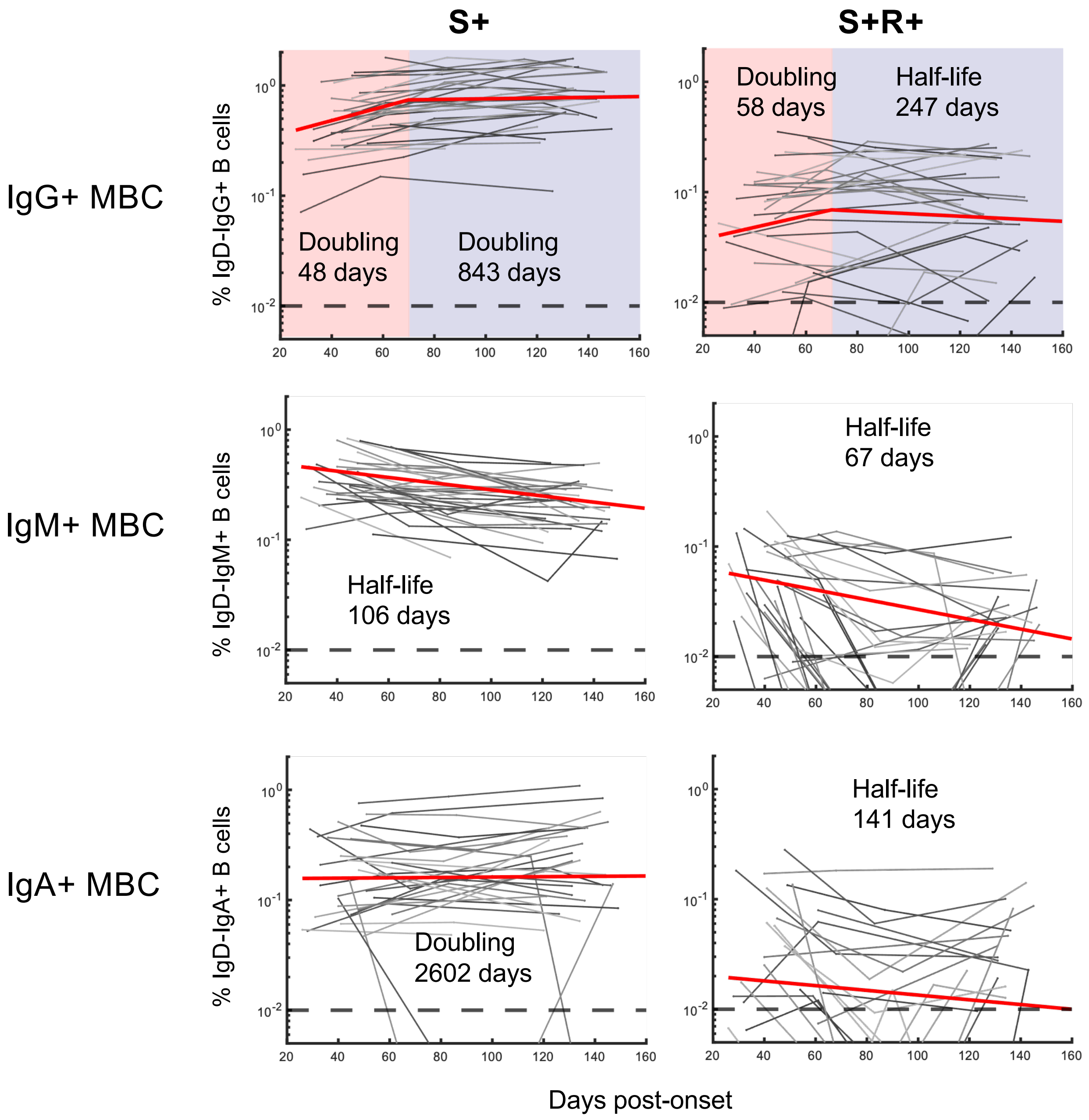

Supplementary Figure 7: Fitting of the kinetics of S- and RBD-specific memory B cell responses over time.

The best-fit half-lives are shown for the fitting of the growth and/or decay of S- or RBD-specific memory B cells (n=31 participants). Two-phase decay is indicated by red (before day 70) and blue (after day 70) shaded areas. No shading indicates where a single-phase decay model was used to fit the data. Dashed line indicates the lower limit of detection. Source data are provided as a Source Data file.

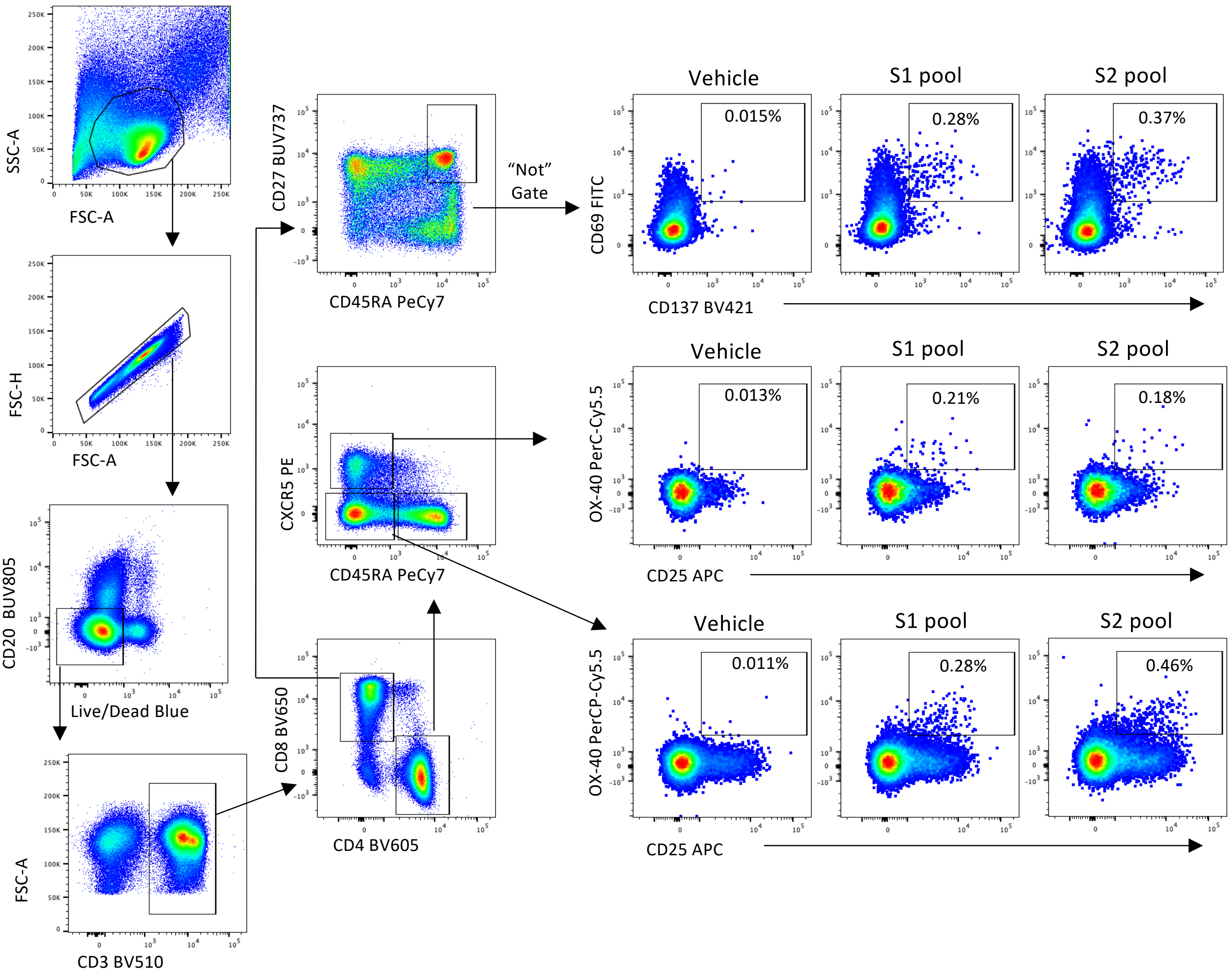

Supplementary Figure 8: Gating strategy for quantifying antigen-specific T cells. Lymphocytes were identified by FSC/SSC, followed by doublet exclusion (FSC-A vs FSC-H), and exclusion of dead or CD20+ cells. After gating on CD3, single positive CD4 or CD8 T cell subsets were identified. CD8 Tmem were gated as non-naïve (CD27+CD45RA+) cells, and assessed for co-expression of CD69 and CD137 following stimulation. CD4 T cells were gated as cTFH (CXCR5+CD45RA-) or Tmem (CXCR5-CD45RA-), and assessed for co-expression of OX-40 and CD25 following stimulation.

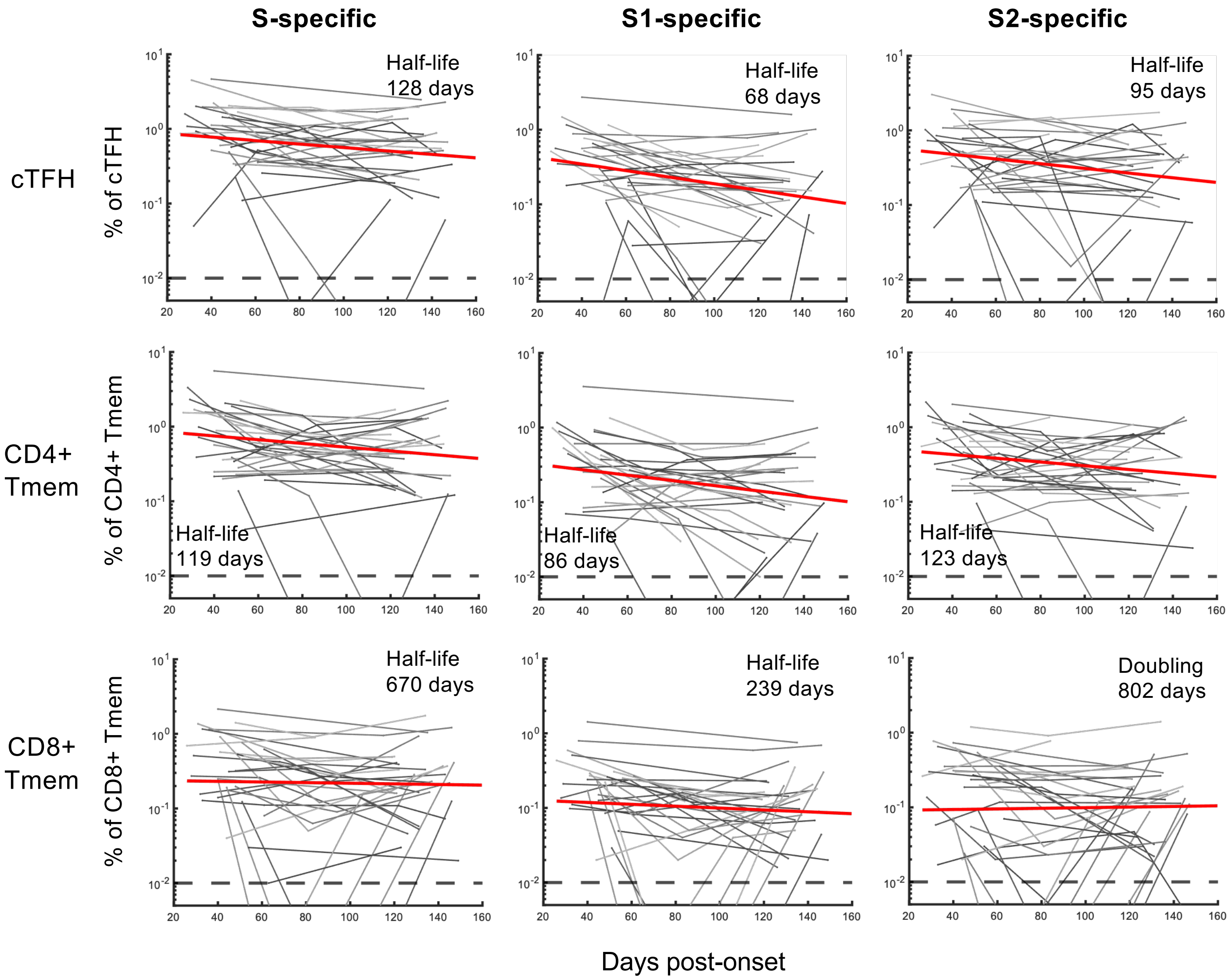

Supplementary Figure 9: Fitting of the decline in SARS-CoV-2-specific T cells over time.

The best-fit half-lives are shown for the fitting of the decay of cTFH, CD4+ Tmem and CD8+ Tmem specific to total S (S1+S2 responses combined), S1 or S2 peptide pools (n=31 participants). In all cases decay was fit with a single-phase decay model with the half-lives shown. Dashed line indicates the lower limit of detection. Source data are provided as a Source Data file.

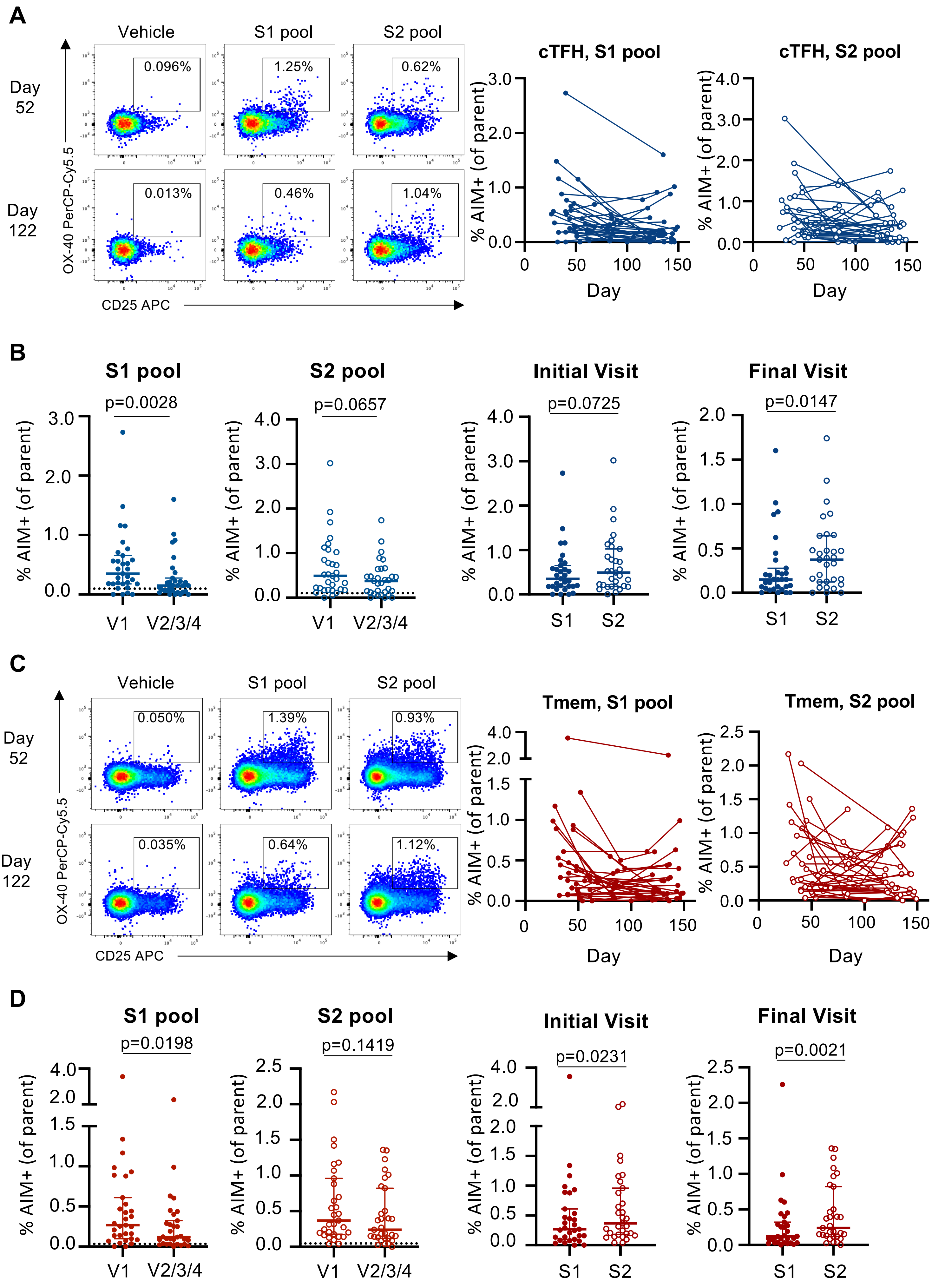

Supplementary Figure 10: S1 and S2-specific CD4+ T cell responses.

Supplementary Figure 10: S1 and S2-specific CD4+ T cell responses.

(**A, C**) Representative staining of AIM markers following S1 and S2 peptide pool stimulation among (**A**) cTFH (CD3+CD4+CD8-CD45RA-CXCR5+) or (**C**) CD4+ Tmem cells and longitudinal cohort analysis (n=31). (**B, D**) Comparison of S1 or S2-specific (**B**) cTFH or (**D**) CD4+ Tmem responses at the earliest and latest visit for each participant, as well as paired frequency of S1 versus S2 responses at the initial or final visit (n=31). All graphs display background-subtracted values. Dashed line indicates median antigen-specific response of n=20 uninfected controls. Statistics assessed by two-tailed Wilcoxon test. Data are shown as median with interquartile range. Source data are provided as a Source Data file.

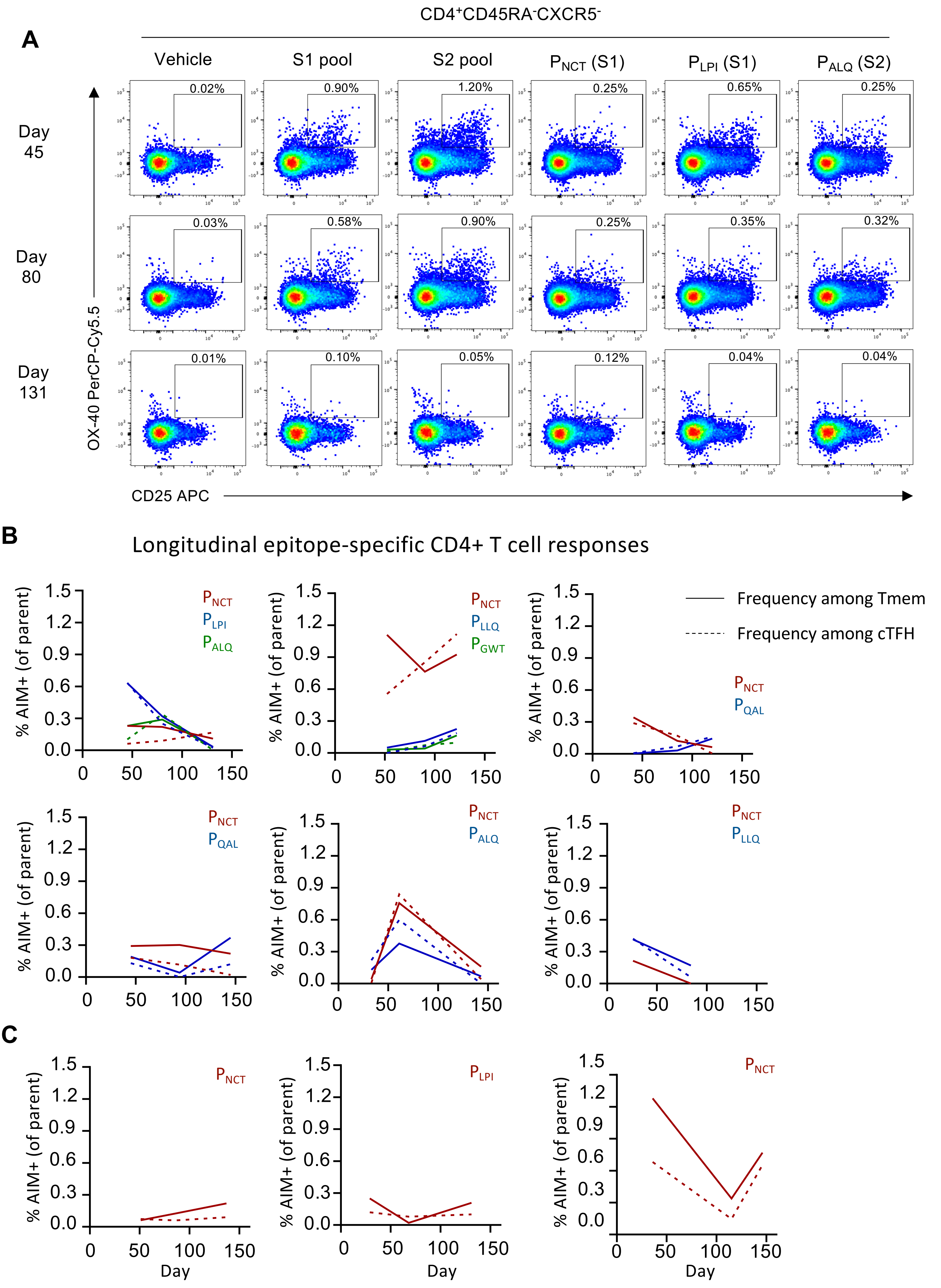

Supplementary Figure 11: Epitope-specific CD4+ T cell responses.

Supplementary Figure 11: Epitope-specific CD4+ T cell responses.

(**A**) Representative staining of AIM markers following S1 or S2 peptide pool or individual peptide stimulation among the CD4+ Tmem population. (**B,C**) Longitudinal peptide-specific frequencies in individual participants (n=9; solid line, CD4+ Tmem; dashed line, cTFH) for whom (**B**) multiple or (**C**) single epitopes were identified. All graphs show background subtracted data. Source data are provided as a Source Data file.

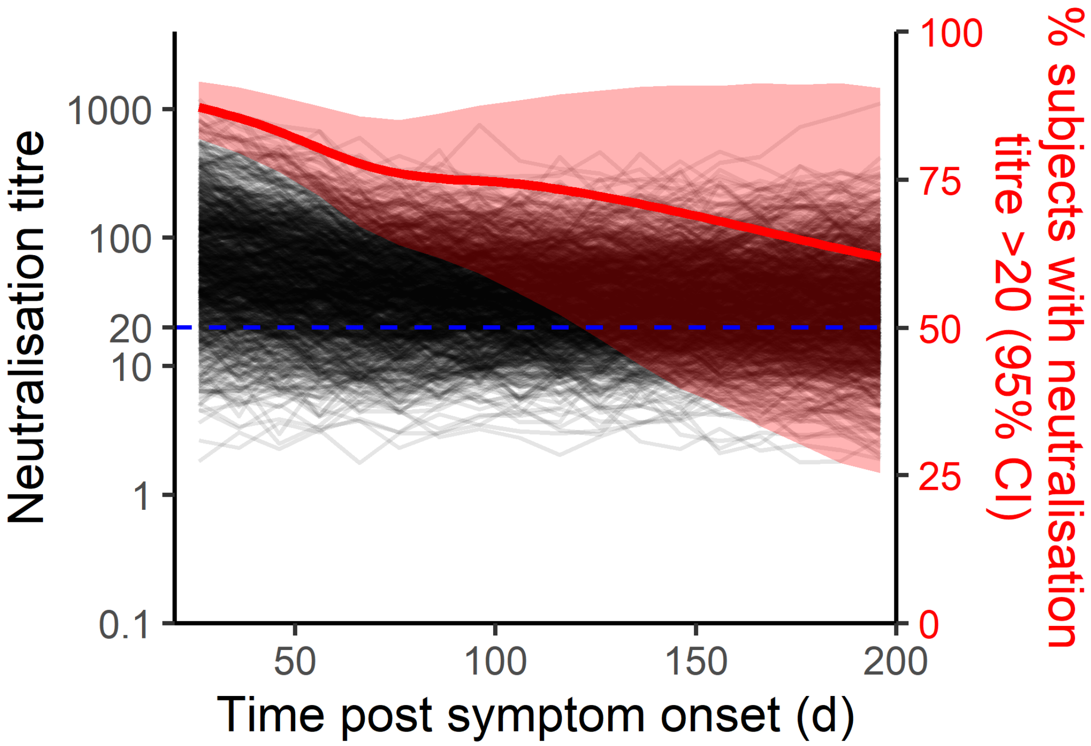

Supplementary Figure 12: Decay of neutralising antibody response to a titre of 1:20. Simulation of elicitation and decay of serological neutralisation activity in 1000 individuals based on distributions observed in our SARS-CoV-2 convalescent cohort. The simulation was repeated 1000 times to estimate the proportion of individuals maintaining a neutralisation titre above 1:20 across multiple simulations (median and 95% confidence intervals shown in red)
