# Supplementary material for: Evolution of immune responses to SARS-CoV-2 in mild-moderate COVID-19
Source: Nat Commun. 2021 Feb 19;12:1162. doi: 10.1038/s41467-021-21444-5 (PMC7896046; doi:10.1038/s41467-021-21444-5)
Supplement: Supplementary file 3 — Reporting Summary [file 41467_2021_21444_MOESM3_ESM.pdf]

nature research

Corresponding author(s): Kent, SJ

Last updated by author(s): Dec 22, 2020

Reporting Summary

Nature Research wishes to improve the reproducibility of the work that we publish. This form provides structure for consistency and transparency in reporting. For further information on Nature Research policies, see our Editorial Policies and the Editorial Policy Checklist.

Statistics

For all statistical analyses, confirm that the following items are present in the figure legend, table legend, main text, or Methods section.

n/a	Confirmed	
[ ]	[x]	The exact sample size (*n*) for each experimental group/condition, given as a discrete number and unit of measurement
[ ]	[x]	A statement on whether measurements were taken from distinct samples or whether the same sample was measured repeatedly
[ ]	[x]	The statistical test(s) used AND whether they are one- or two-sided<br>*Only common tests should be described solely by name; describe more complex techniques in the Methods section.*
[ ]	[x]	A description of all covariates tested
[ ]	[x]	A description of any assumptions or corrections, such as tests of normality and adjustment for multiple comparisons
[ ]	[x]	A full description of the statistical parameters including central tendency (e.g. means) or other basic estimates (e.g. regression coefficient) AND variation (e.g. standard deviation) or associated estimates of uncertainty (e.g. confidence intervals)
[ ]	[x]	For null hypothesis testing, the test statistic (e.g. *F*, *t*, *r*) with confidence intervals, effect sizes, degrees of freedom and *P* value noted<br>*Give P values as exact values whenever suitable.*
[x]	[ ]	For Bayesian analysis, information on the choice of priors and Markov chain Monte Carlo settings
[ ]	[x]	For hierarchical and complex designs, identification of the appropriate level for tests and full reporting of outcomes
[ ]	[x]	Estimates of effect sizes (e.g. Cohen's *d*, Pearson's *r*), indicating how they were calculated

Our web collection on statistics for biologists contains articles on many of the points above.

Software and code

Policy information about availability of computer code

Data collection	BD FACS Diva v9.0, FlowJo v10, Peaks Studio version X-plus
Data analysis	All data analysis, fitting, statistical tests and bootstrapping were performed using the statistical soft R (version 4.0.2) and associated libraries (in particular, censReg v0.5-30, lmec v1.0), and GraphPad Prism 8. All data analysis code can be made available on request.

For manuscripts utilizing custom algorithms or software that are central to the research but not yet described in published literature, software must be made available to editors and reviewers. We strongly encourage code deposition in a community repository (e.g. GitHub). See the Nature Research guidelines for submitting code & software for further information.

Data

Policy information about availability of data

All manuscripts must include a data availability statement. This statement should provide the following information, where applicable:

- Accession codes, unique identifiers, or web links for publicly available datasets
- A list of figures that have associated raw data
- A description of any restrictions on data availability

Data are provided in the Source Data file and any queries on the data will be attended to by the corresponding author upon reasonable request.

Field-specific reporting

Life sciences study design

All studies must disclose on these points even when the disclosure is negative.

Sample size	The sample size was based on the recruitment of individuals for whom more than 1 longitudinal plasma or PBMC sample was available for analysis, providing 158 individual data points. This sample size is comparable and larger and with a longer follow-up than most similar studies.
Data exclusions	No data were excluded from analysis.
Replication	Serological assays included technical replicates (duplicate or quadruplicate) on each assay plate, as well as independent technical replicates performed on different days. All attempts at replication or serological assays were successful. Assays of cellular immunity were not replicated owing to limiting cell numbers.
Randomization	There was no grouping of participants in the study
Blinding	The investigators were blinded to group allocation during data collection.

Reporting for specific materials, systems and methods

We require information from authors about some types of materials, experimental systems and methods used in many studies. Here, indicate whether each material, system or method listed is relevant to your study. If you are not sure if a list item applies to your research, read the appropriate section before selecting a response.

Materials & experimental systems

n/a	Involved in the study	
☐	☒	Antibodies
☐	☒	Eukaryotic cell lines
☒	☐	Palaeontology and archaeology
☒	☐	Animals and other organisms
☐	☒	Human research participants
☒	☐	Clinical data
☒	☐	Dual use research of concern

Methods

n/a	Involved in the study	
☒	☐	ChIP-seq
☐	☒	Flow cytometry
☒	☐	MRI-based neuroimaging

Antibodies

Antibodies used	T cells were stained with: stained with Live/dead Blue viability dye (ThermoFisher), CD27 BUV737 (L128), , CD45RA PeCy7 (HI100), CD20 BUV805 (2H7), (BD Biosciences), CD3 BV510 (SK7), CD4 BV605 (RPA-T4), CD8 BV650 (RPA-T8), CD25 APC (BC96), OX-40 PerCP-Cy5.5 (ACT35), CD69 FITC (FN50), CD137 BV421 (4B4-1) (Biolegend), and CXCR5 PE (MU5UBEE, ThermoFisher).<br>B cells were stained with: Aqua viability dye (Thermofisher), CD19-ECD (J3-119) (Beckman Coulter), CD20 Alexa700 (2H7), IgM-BUV395 (G20-127), CD21-BUV737 (B-ly4), IgD-Cy7PE (IA6-2), IgG-BV786 (G18-145) (BD), CD14-BV510 (M5E2), CD3-BV510 (OKT3), CD8a-BV510 (RPA-T8), CD16-BV510 (3G8), CD10-BV510 (HI10a), CD27-BV605 (O323) (Biolegend), IgA-Vio450 (clone) (Miltenyi), Anti-SARS-CoV-2 RBD neutralising human IgG1 (SAD-S35, Acrobiosystems), biotinylated mouse anti-human IgM (mAb MT22; MabTech) .
Validation	All antibodies were commercially validated for use in flow cytometry on human cells by the manufacturers Thermofisher, BD Biosciences, Biolegend, Beckman Coulter, Miltenyi, Acrobiosystems and Mabtech. Full validation statements available on manufacturer's websites.

Eukaryotic cell lines

Policy information about cell lines

Cell line source(s)	Expi293F cells were obtained from ThermoFisher (#A14527)
Authentication	None of the cell lines used were authenticated
Mycoplasma contamination	Expi293 cells were not tested for mycoplasma.
Commonly misidentified lines (See ICLAC register)	No commonly misidentified cell lines were used during the study.

Human research participants

Policy information about studies involving human research participants

Population characteristics	The cohort was a median of 55 years old and 44% female. The characteristics are reported in Supplementary Fig 1.
Recruitment	Participants were recruited by contact with the study investigators, and through word of mouth among SARS-CoV-2 infection clusters. Participants may have self-selected for people with a greater interest in COVID-19 infection, but this is unlikely to affect the results of the study.
Ethics oversight	The study protocols were approved by the University of Melbourne Human Research Ethics Committee (#2056689) and the Southern Adelaide Clinical Human Research Ethics Committee (#39.034), and all associated procedures were carried out in accordance with the approved guidelines. All participants provided written informed consent in accordance with the Declaration of Helsinki.

Note that full information on the approval of the study protocol must also be provided in the manuscript.

Flow Cytometry

Plots

Confirm that:

- [x] The axis labels state the marker and fluorochrome used (e.g. CD4-FITC).
- [x] The axis scales are clearly visible. Include numbers along axes only for bottom left plot of group (a 'group' is an analysis of identical markers).
- [x] All plots are contour plots with outliers or pseudocolor plots.
- [x] A numerical value for number of cells or percentage (with statistics) is provided.

Methodology

Sample preparation	PBMC were isolated from whole blood. Samples were cryopreserved, and thawed and stained in batches. For antigen-specific T cell assays, cells were stained after stimulation.
Instrument	Samples were acquired on a BD LSR Fortessa or BD Aria II.
Software	Samples were acquired using BD FACS Diva, and analysed using FlowJo version 10
Cell population abundance	Cell population abundance was 10e6 PBMC per well.
Gating strategy	After doublet exclusion (FSC-A vs FSC-H) and lymphocyte gating (FSC-A vs SSC-A), live CD19+IgD-CD20+ B cells were gated based on surface immunoglobulin expression (IgM, IgG, IgA). Binding to SARS-CoV-2 spike (S) and/or SARS-CoV-2 RBD probes was assessed for each population. Memory B cell phenotypes were identified by CD21 and CD27 co-staining.<br>Lymphocytes were identified by FSC/SSC, followed by doublet exclusion (FSC-A vs FSC-H), and exclusion of dead or CD20+ cells. After gating on CD3, single positive CD4 or CD8 T cell subsets were identified. CD8 Tmem were gated as non-naïve (CD27+CD45RA+) cells, and assessed for co-expression of CD69 and CD137 following stimulation. CD4 T cells were gated as cTFH (CXCR5+CD45RA-) or Tmem (CXCR5-CD45RA-), and assessed for co-expression of OX-40 and CD25 following stimulation.

- [x] Tick this box to confirm that a figure exemplifying the gating strategy is provided in the Supplementary Information.
